# Long-Term Outcomes Following Conventionally Fractionated Stereotactic Boost for High-Grade Gliomas in Close Proximity to Critical Organs at Risk

**DOI:** 10.3389/fonc.2018.00373

**Published:** 2018-09-11

**Authors:** Michael C. Repka, Siyuan Lei, Lloyd Campbell, Simeng Suy, Jean-Marc Voyadzis, Christopher Kalhorn, Kevin McGrail, Walter Jean, Deepa S. Subramaniam, Jonathan W. Lischalk, Sean P. Collins, Brian T. Collins

**Affiliations:** ^1^Department of Radiation Medicine, MedStar Georgetown University Hospital, Washington, DC, United States; ^2^Department of Neurosurgery, MedStar Georgetown University Hospital, Washington, DC, United States; ^3^Department of Neurological Surgery, George Washington University Hospital, Washington, DC, United States; ^4^Division of Hematology and Oncology, MedStar Georgetown University Hospital, Washington, DC, United States

**Keywords:** stereotactic, Cyberknife, high-grade glioma, glioblastoma, boost, fractionated

## Abstract

**Purpose/Objective:** High-grade glioma is the most common primary malignant tumor of the CNS, with death often resulting from uncontrollable intracranial disease. Radiation dose may be limited by the tolerance of critical structures, such as the brainstem and optic apparatus. In this report, long-term outcomes in patients treated with conventionally fractionated stereotactic boost for tumors in close proximity to critical structures are presented.

**Materials/Methods:** Patients eligible for inclusion in this single institution retrospective review had a pathologically confirmed high-grade glioma status post-surgical resection. Inclusion criteria required tumor location within one centimeter of a critical structure, including the optic chiasm, optic nerve, and brainstem. Radiation therapy consisted of external beam radiation followed by a conventionally fractionated stereotactic boost. Oncologic outcomes and toxicity were assessed.

**Results:** Thirty patients eligible for study inclusion underwent resection of a high-grade glioma. The median initial adjuvant EBRT dose was 50 Gy with a median conventionally fractionated stereotactic boost of 10 Gy. All stereotactic treatments were given in 2 Gy daily fractions. Median follow-up time for the entire cohort was 38 months with a median overall survival of 45 months and 5-year overall survival of 32.5%. The median freedom from local progression was 45 months, and the 5-year freedom from local progression was 29.7%. Two cases of radiation retinopathy were identified following treatment. No patient experienced toxicity attributable to the optic chiasm, optic nerve, or brainstem and no grade 3+ radionecrosis was observed.

**Conclusions:** Oncologic and toxicity outcomes in high-grade glioma patients with tumors in unfavorable locations treated with conventionally fractionated stereotactic boost are comparable to those reported in the literature. This treatment strategy is appropriate for those patients with resected high-grade glioma in close proximity to critical structures.

## Introduction

High-grade glioma is the most common primary malignant tumor of the central nervous system (CNS), and tends to be locally aggressive, with death often resulting from uncontrollable intracranial disease (1). Although several strategies have been employed to improve local control rates in patients with malignant glioma, relatively few have produced meaningful clinical outcomes. Early trials in the 1960s and 1970s from the Brain Tumor Study Group (BTSG) demonstrated a survival benefit for the addition of post-operative radiation following neurosurgical resection (2), but it would be decades before a role for systemic therapy was convincingly established. In 2005, a seminal trial by the European Organization for Research and Treatment of Cancer (EORTC) demonstrated an overall survival benefit for the addition of concomitant and adjuvant temozolomide, but the prognosis for patients with glioblastoma remains grim, with an overall survival rate of <10% at 5 years (3, 4).

Nevertheless, there is a clear subset of patients who achieve prolonged survival following treatment for high-grade glioma. Although the majority of glioblastoma patients will die in the 2 years following diagnosis, the clear tail seen on the survival curves presented by Stupp et al. suggests that cure is attainable for a small group of patients (4). Furthermore, patients with WHO Grade III disease can live for many years, particularly in the setting of a 1p/19q co-deletion (5, 6). Emerging data indicates that microscopic histologic grading, which has served a critical role in determining prognosis and therapy, may be made obsolete as our understanding of underlying tumor genetics evolves (7, 8). For instance, IDH-1 mutational status is critical in the modern WHO grading of both low- and high-grade gliomas (9). However, until these data grow more robust, predicting which patients may expect long-term survival remains challenging. For this reason, care must be taken when planning treatment, given the potential for devastating late toxicities with cranial irradiation.

In 2010, we initially reported oncologic and toxicity outcomes from a cohort of patients with high-grade gliomas located in close proximity to critical intracranial organs (10). In order to minimize the likelihood of long-term toxicity, these patients received a conventionally fractionated stereotactic boost to the tumor cavity and any present residual tumor following initial treatment with external beam radiotherapy (EBRT). In this report, we present the long-term oncologic and toxicity outcomes in this cohort of patients.

## Methods and materials

### Patient selection

The Georgetown University Institutional Review Board (IRB) approved this single institution retrospective study. Patients eligible for study inclusion had a pathologically confirmed high-grade glioma (2007 WHO Grade III-IV) status post-surgical resection who were deemed candidates for post-operative cranial radiotherapy. Study criteria for patient inclusion consisted of age at diagnosis greater than 15 years, post-operative Eastern Cooperative Oncology Group (ECOG) performance status (PS) of 0–1, and tumor location within one centimeter of a critical structure. Critical structures included the optic chiasm, optic nerve, and brainstem. Given the particularly poor prognoses following biopsy without any resection (11), these patients were excluded from the study.

### Radiation treatment planning and delivery

Patients were simulated with a custom thermoplastic mask in the supine position. All patients underwent high-resolution computed tomography (CT) scan with a minimum of 1.25 mm slice thickness. Pre- and post-operative MRI scans were fused to the planning CT scan in order to help delineate residual tumor if applicable, the resection cavity, and areas at risk for microscopic spread. Target volumes and radiation fields were designed at the discretion of the treating radiation oncologist with neurosurgical input; no standardized RTOG or EORTC volumes were employed. Conventional radiation therapy consisted of either 3D conformal radiation therapy (3D-CRT) or intensity modulated radiation therapy (IMRT) to an initial dose of 44–50.4 Gy to the residual tumor if applicable, the resection cavity, and areas at risk for microscopic spread. This was followed by a conventionally fractionated stereotactic boost to the resection cavity and residual tumor using the CyberKnife (Accuray Inc., Sunnyvale, CA, USA) robotic stereotactic radiosurgical system with six-dimensional skull tracking to a total dose of 54–60 Gy. Organs-at-risk (OAR) were limited per dose objectives as outlined in Table 1. Concurrent chemotherapy was given at the discretion of the attending medical oncologist, although all patients after 2004 received concurrent and adjuvant temozolomide. Usage, type, and duration of adjuvant therapy was administered at the discretion of the attending medical oncologist.

**Table 1:** Critical structure radiation dose constraints.

**Critical structure**	**Objective (Gy)**
Lens	10
Retina	50
Optic nerve	55
Optic chiasm	55
Brainstem	55

### Follow-up and statistical analysis

In general, patients underwent clinical evaluation and surveillance MRI of the brain every 3–6 months for 5 years unless local progression or death occurred prior to that time. Late toxicity was scored according to the National Cancer Institute Common Terminology Criteria for Adverse Events, Version 3.0 (NCI-CTCAE 3.0). Local failure was scored according to the Response Assessment in Neuro-Oncology (RANO) Criteria (12). Follow-up time, overall survival, and freedom from local progression were determined from the date of surgery. Overall survival and freedom from local progression estimates were calculated using the Kaplan-Meier method. Statistically significant predictors of overall survival and freedom from local progression were identified on univariate analysis using the log-rank test and Cox proportional hazards model. Multivariable regression analysis was performed using the Cox proportional hazards model to confirm statistically significant predictors of overall survival and freedom from local progression. Statistical analysis was performed using SPSS Statistics version 24.0 (IBM Corporation, Armonk, NY, USA).

## Results

### Patient and treatment characteristics

Between September 2002 and April 2012, 30 patients eligible for study inclusion underwent resection of a high-grade glioma. Baseline patient and tumor characteristics are summarized in Table 2. The median age at diagnosis was 56.5 years, with a range from 16 to 72 years. Twenty-three patients were Caucasian, five were African-American, and one patient was Hispanic. Seventeen patients were male and 13 patients were female. Twenty-eight patients were classified as ECOG performance status 0, while the remaining two patients were classified as ECOG performance status 1. Twelve tumors were located in the frontal lobe, 16 tumors were located in the temporal lobe, one tumor was located in the parietal lobe, and one tumor was located in the occipital lobe.

**Table 2:** Baseline patient and tumor characteristics.

	**Number**	**Percentage (%)**	**Median (Range)**
**Age (years)**			56.5 (16–72)
**Total radiation dose (Gy)**			60 (54–60.4)
**MIB-1 index (%)**			25 (5–66)
**Ethnicity**			
Caucasian	23	76.7	
African-American	5	16.7	
Other	1	3.3	
**Gender**			
Male	17	56.7	
Female	13	43.3	
**ECOG performance status**			
0	28	93.3	
1	2	6.7	
**Histology**			
Grade III	17	56.7	
Grade IV	13	43.3	
**Extent of resection**			
Total	21	70.0	
Subtotal	9	30.0	
**Lobe treated**			
Temporal	16	53.3	
Frontal	12	40.0	
Parietal	1	3.3	
Occipital	1	3.3	

Twenty-one patients underwent a gross total resection (GTR) of their tumor, while the remaining nine underwent subtotal resection (STR). Carmustine wafers (GLIADEL, Arbor Pharmaceuticals LLC, Atlanta, GA, USA) were implanted at the time of resection in two patients. Glioblastoma (WHO Grade IV) was identified in the surgical pathology specimens of 13 patients, while the remaining 17 patients were classified as having WHO Grade III disease. The median MIB-1 proliferative index was 25%, with a range of 5–66%. The MGMT methylation status was only reported in two patients, and was positive in one patient. Of the 17 patients with Grade III disease, a 1p/19q co-deletion was reported in five patients (29.4%).

All patients received post-operative EBRT followed by conventionally fractionated robotic stereotactic boost. The first phase of post-operative EBRT was delivered using 3D-CRT in the majority of patients; however, 2 patients received IMRT. The median post-operative EBRT dose in the first treatment phase was 50 Gy, with a range from 44 to 50.4 Gy. The median conventionally fractionated stereotactic boost was 10 Gy, with a range of 10–14 Gy, and all stereotactic treatments were given in 2 Gy daily fractions. For the boost phase, the median prescription isodose line was 72% with a range from 67 to 80%. Concurrent chemotherapy was given to 24 patients (80%), all of whom received temozolomide. Adjuvant chemotherapy was administered to all patients. 28 patients received temozolomide, while the remaining 2 patients received a combination of procarbazine, lomustine, and vincristine (PCV).

### Oncologic outcomes

**Figure 1:**
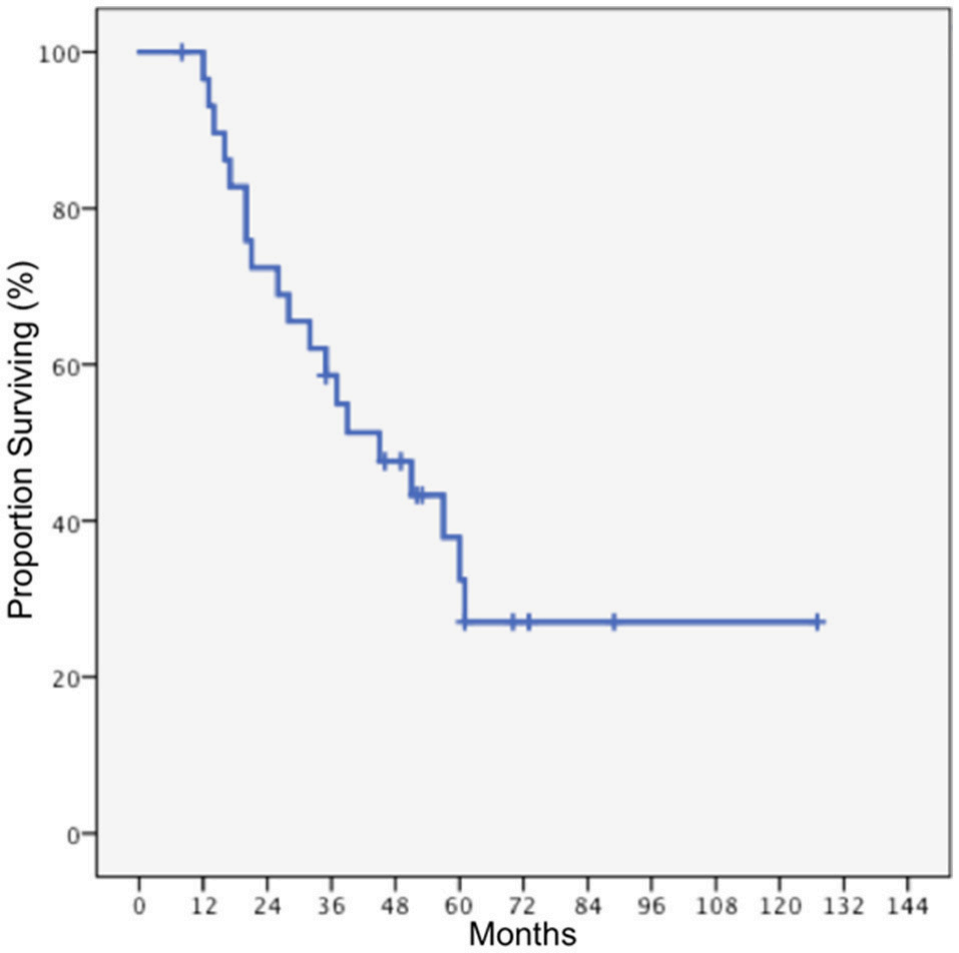
Overall survival for the entire cohort.

**Figure 2:**
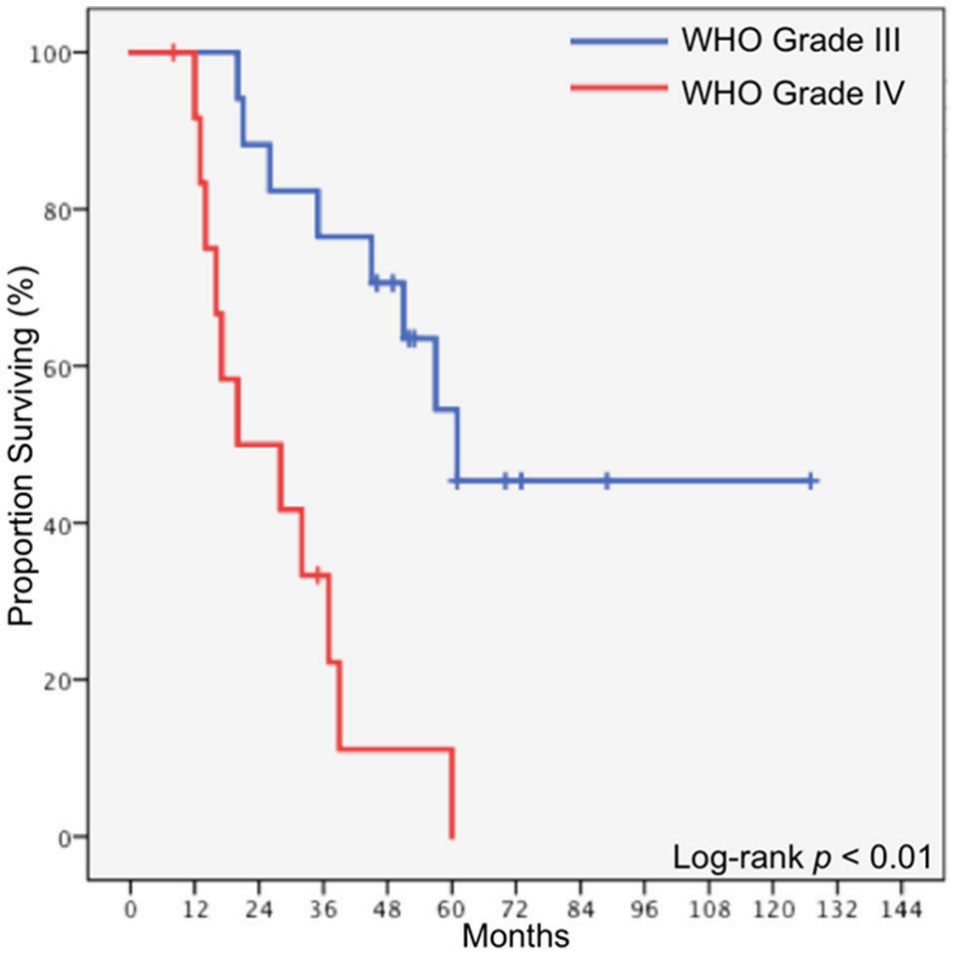
Overall survival stratified by histology.

Median follow-up time for the entire cohort was 38 months, with a range of 8 months to 127 months. The median overall survival for the entire cohort was 45 months, and the 5-year overall survival was 32.5% (Figure 1). Local tumor progression was the cause of death in 80% of patients who died, while two patients died of pulmonary embolism, one died of cerebrovascular accident, and one died of extracranial disease progression (spinal leptomeningeal metastases). Eleven patients underwent salvage surgery, and in each case recurrent disease was confirmed upon pathologic review. Two patients received salvage radiation, using either single dose or fractionated radiosurgery. In patients who underwent GTR, the median overall survival was 57 months, compared to 20 months in patients who underwent STR (*p* = 0.015). In patients with WHO Grade III disease, the median overall survival was 61 months, compared to 20 months in patients with glioblastoma (*p* < 0.01, Figure 2).

**Figure 3:**
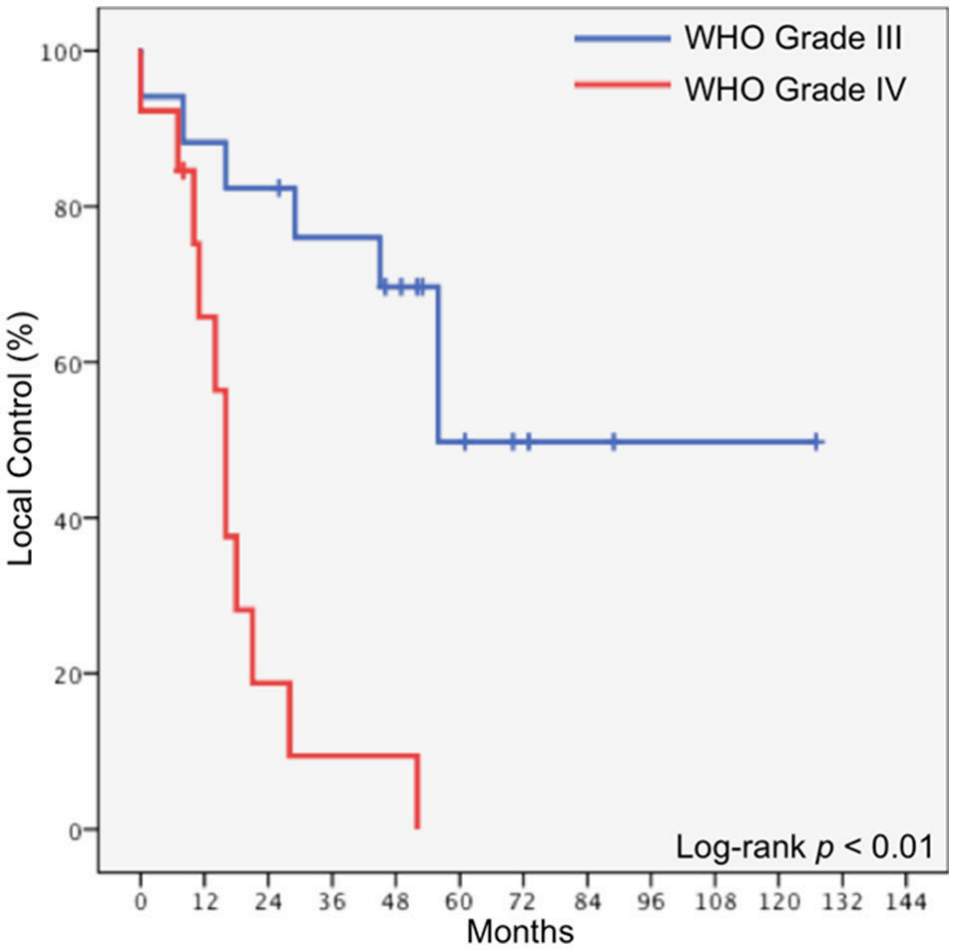
Local control stratified by histology.

The median freedom from local progression was also 45 months, and the 5-year freedom from local progression was 29.7%. In patients who underwent GTR, the median freedom from local progression was 56 months, compared to 16 months in patients who underwent STR (*p* = 0.015). In patients with WHO Grade III disease, the median freedom from local progression was 56 months, compared to 16 months in patients with glioblastoma (*p* < 0.01, Figure 3).

On univariate analysis, histology and extent of tumor resection were significant predictors of both overall survival and freedom from local progression (Table 3). Patient age, MIB-1 proliferative index, ECOG PS, and tumor location were not found to be statistically significant predictors of oncologic outcomes. Multivariable testing confirmed tumor histology to be an independent predictor of overall survival (HR = 0.200, 95% CI 0.073–0.549, *p* = 0.002) and freedom from local progression (HR = 0.153, 95% CI 0.047–0.496, *p* = 0.002), while extent of resection was not confirmed as a statistically significant predictor of either outcome (Table 4).

**Table 3:** Overall survival.

	**Univariate analysis**			**Multivariable analysis**		
	**HR**	**95% CI**	***p***	**HR**	**95% CI**	***P***
Age	1.030	0.996–1.064	0.081	–	–	–
Histology	0.187	0.069–0.506	<0.01	0.200	0.073–0.549	<0.01
MIB-1	5.446	0.415–71.436	0.197	–	–	–
Resection	2.578	1.002–6.631	0.049	2.189	0.828–5.79	0.114

**Table 4:** Local control.

	**Univariate analysis**			**Multivariable analysis**		
	**HR**	**95% CI**	***p***	**HR**	**95% CI**	***P***
Age	1.032	0.997–1.069	0.071	–	–	–
Histology	0.370	0.210–0.651	<0.01	0.153	0.047–0.496	<0.01
MIB-1	5.521	0.392–77.812	0.206	–	–	–
Resection	3.146	1.172–8.446	0.023	2.221	0.773–6.38	0.138

### Toxicity

Overall, treatment was well tolerated with minimal appreciable toxicity. One patient was found to have radiographic evidence of necrosis on follow-up MRI, but subsequent repeat craniotomy confirmed the presence of recurrent disease. Five patients (17.0%) experienced radiographic pseudoprogression on MRI. No patient experienced toxicity attributable to optic chiasm, optic nerve, or brainstem damage. Of the 11 patients who underwent salvage surgery, all patients had evidence of recurrent tumor. No cases of grade 3+ radiation necrosis were identified.

Two patients with WHO Grade III tumors developed retinopathy following treatment. One patient, who presented initially with a left frontal lobe tumor, suffered from bilateral retinal hemorrhage 8 months following completion of radiation which required laser photocoagulation and intraocular bevacizumab. The initial radiation treatment consisted of opposed lateral 3D-CRT beam arrangement, with the bilateral eyes receiving <25 cGy from the stereotactic boost. This patient remains alive with functional vision at the time of last follow-up. The second patient had a right fronto-temporal tumor and developed right radiation retinopathy which was unresponsive to laser photocoagulation. This patient was treated with a vertex and lateral field arrangement, with the boost phase contributing ~500 cGy to the eye. Second line bevacizumab was effective, and at last follow-up the patient was alive with a mild visual acuity deficit in the right eye.

## Discussion

High-grade glioma is a challenging illness, with a disease course dominated by progressive neurocognitive decline and eventual death for most patients. Although intracranial disease progression is typical, efforts to improve and maintain local control have resulted in relatively small gains for these patients. Certain surgical techniques, such as the use of 5-aminolevulinic acid, may increase the rate of gross total resection and progression free survival, (13) but microscopic disease is always left behind within the peri-tumoral edema (14). Furthermore, the infiltrative nature of glioblastoma acts in concert with the high-density of critical healthy tissue within the brain to limit the extent of many operative interventions.

Efforts to improve local control following surgery have been equally uninspiring. Although there is some evidence to suggest that implantable carmustine wafers may improve survival, their efficacy is controversial, they can cause significant toxicity, and their role is unclear in the temozolomide era (15). Alternative therapies, such as electromagnetic tumor treating fields, may yet hold the key to better disease control but appear to extend survival only by a few months (16). Beyond temozolomide, other systemic therapies, such as the anti-angiogenic drug bevacizumab, have had no impact on survival rates (17, 18). Finally, attempts to increase radiation dose above 60 Gy have been generally unrewarding since the BTSG published its seminal paper on dose-response in 1979 (19). For example, a group from the University of Michigan attempted radiation doses as high as 90 Gy, producing high rates of radionecrosis without a significant improvement in local failure rates over historical data (20). Nonetheless, dose escalation for glioma remains a holy grail of clinical radiation research, as evidenced by the multiple ongoing phase III trials including NRG BN-001 and INTRAGO-II (21, 22).

In spite of these persistent challenges, there remains a subset of patients who achieve durable local control, extended survival, and in some cases even cure. For these patients, the long-term consequences of craniotomy followed by chemoradiation may be devastating. Currently, there is no universal standard of care for target delineation in patients with high-grade glioma. The EORTC recommends a smaller volume approach, treating a single volume to 60 Gy as performed in the landmark trial reported by Stupp et al. In the United States, however, the NRG recommends a more generous sequential approach that more comprehensively covers areas of suspected microscopic spread (14, 23). Secondary analyses of two phase III randomized trials have not identified a difference between these treatment approaches with regards to survival or patterns of failure (24, 25). Although only published in abstract form, there is randomized evidence that suggests larger volumes may be associated with a detriment in overall survival (26). While the optimal method for delivering post-operative chemoradiation remains unclear, the importance of respecting normal tissue tolerance should not be casually disregarded.

In this study, we report long-term outcomes of patients treated with a conventionally fractionated stereotactic boost for high-grade glioma. This treatment was well tolerated, with oncologic outcomes similar to those reported in the seminal studies. Furthermore, there was minimal long-term toxicity associated with post-operative radiation, although two patients did suffer from retinopathy requiring intervention. Encouragingly, we were unable to identify a confirmed case of radionecrosis in any of these patients treated with a conventionally fractionated stereotactic boost.

Some limitations of this study preclude recommending such a treatment strategy to all patients with high-grade glioma. First, the study was limited to a single institution, employed a relatively small cohort, and included only patients with target volumes close to critical structures. Additionally, given the recent advances in the general understanding of glioma genetics, this study lacks information on these tumors' molecular subtypes. Furthermore, recent advances in IMRT and volumetric modulated arc therapy (VMAT) may have reduced the need for a fractionated stereotactic boost in some patients. However, the recommended minimum PTV margin of 3–5 mm with these strategies (27) coupled with the ongoing interest in dose escalation, might suggest a larger future role for the fractionated stereotactic boost approach reported in this study.

## Conclusions

While the optimal therapy for high-grade glioma remains undiscovered, concurrent chemoradiation with temozolomide to a total dose of 60 Gy is the most appropriate initial adjuvant treatment for appropriately selected patients. Tumors in close proximity to critical structures, particularly those seated deep in the temporal or frontal lobes, may be difficult to adequately cover with conventional linear accelerator based radiation delivery. Our data suggest that in patients with tumors in close proximity to critical structures, a conventionally fractionated, image-guided stereotactic boost allows for optimal dose delivery with an excellent long-term toxicity profile.

## Ethics statement

This study was carried out in accordance with the recommendations of the Georgetown University Institutional Review Board (IRB). The protocol was approved by the Georgetown University Institutional Review Board (IRB). All subjects gave written informed consent in accordance with the Declaration of Helsinki.

## Author contributions

MR wrote the manuscript. MR, SL, LC, SC, and BC extracted the data and created the database. MR, SS, and BC performed the data analysis. MR, SS, J-MV, CK, KM, WJ, JL, DS, SC, JL, and BC reviewed the data analysis and study conclusions. BC edited the manuscript and approved the final draft.

### Conflict of interest statement

SC and BC are clinical consultants for Accuray, Inc. The remaining authors declare that the research was conducted in the absence of any commercial or financial relationships that could be construed as a potential conflict of interest.
